# Dependence of Left Ventricular Rotational Mechanics on Left Atrial Volumes in Non-Smoker Healthy Adults: Analysis Based on the Three-Dimensional Speckle-Tracking Echocardiographic MAGYAR-Healthy Study

**DOI:** 10.3390/jcm12031235

**Published:** 2023-02-03

**Authors:** Attila Nemes, Árpád Kormányos, Zoltán Ruzsa, Alexandru Achim, Nóra Ambrus, Csaba Lengyel

**Affiliations:** Department of Medicine, Albert Szent-Györgyi Medical School, University of Szeged, 6725 Szeged, Hungary

**Keywords:** left ventricular, rotation, left atrial, volume, three-dimensional, echocardiography

## Abstract

**Introduction**: As has been established, the left ventricle (LV) and the left atrium (LA) form an organic unit of the left heart; however, little is known about the dependence of LV rotational parameters on LA volumes, even in healthy circumstances. Therefore, the present study aimed to assess the associations between basal and apical LV rotations and LA volumes and volume-based functional properties throughout the cardiac cycle in healthy adults by three-dimensional speckle-tracking echocardiography. **Methods**: The present study comprised 167 healthy adults (age: 33.4 ± 12.6 years, 77 males) with normally directed LV rotational mechanics. All subjects underwent complete two-dimensional Doppler echocardiography with three-dimensional speckle-tracking echocardiography (3DSTE)-derived data acquisition. The 3DSTE-derived LA volumes and LV rotational parameters were determined at a later date. **Results**: An increasing end-systolic maximum LA volume (Vmax) was associated with increasing pre-atrial-contraction early (VpreA) and minimum end-diastolic (Vmin) LA volumes, and all stroke volumes were increased as well. Systolic basal left ventricular rotation (LVrot) was highest in the case of the highest systolic Vmax and early-diastolic VpreA. Apical LVrot did not show obvious associations with any increasing LA volumes. The highest systolic basal LVrot was associated with significantly increased diastolic VpreA and Vmin. Reduced diastolic LA volumes (VpreA, Vmin) were seen in the case of increased apical LVrot. An increasing basal LVrot was associated with the tendentious lowering of the apical LVrot and the significant elevation of LV twist. Similarly, an increasing apical LVrot was associated with the tendentious lowering of basal LVrot and the significant elevation of LV twist. **Conclusions**: Strong associations and adaptations between 3DSTE-derived LA volumes throughout the cardiac cycle and LV rotational mechanics were evidenced, even in healthy circumstances.

## 1. Introduction

Novel cardiovascular imaging techniques enable the detailed morphologic and functional assessment of the heart and its components. Moreover, the simultaneous creation of virtual three-dimensional (3D) models of certain cardiac chambers from a dataset acquired at the same time could help us understand how vessels, ventricles, atria, and valves (and their annuli) affect each other even in healthy conditions. Three-dimensional speckle-tracking echocardiography (STE) is one of the best examples of these modalities. It is an easy-to-learn/easy-to-use cardiovascular imaging tool with the ability to provide a detailed analysis of the volumes, strains, and rotational mechanics of certain cardiac chambers, together with the spatial, morphological, and functional analysis of valves [1,2,3,4]. In normal circumstances, the left ventricular (LV) base and apex rotate in opposite directions, resulting in a towel-wringing-like movement called an LV twist [5,6]. Until the development of 3DSTE, LV rotational mechanics were difficult to examine, as only expensive and invasive methods were able to quantify LV segmental/regional rotational movements [6]. With 3DSTE, LV basal and apical segments rotating in opposite directions could be measured in a non-invasive way.

As is already known, the LV and the left atrium (LA) form an organic unit of the left heart. There is relationship between preload, myocardial performance, and cardiac output, with LV stroke volume (SV) depending on LV end-diastolic volume, which is related to LA volume via the Frank–Starling law [7,8]. Although LV rotational mechanics play a significant role in ejection [6], little is known about the dependence of LV rotational parameters determined using cardiac imaging on LA volumes, even in healthy circumstances. Therefore, the present study aimed to assess associations between LV basal and apical rotations and LA volumes and volume-based functional properties throughout the cardiac cycle in healthy adults by 3DSTE.

## 2. Subjects and Methods

### 2.1. Subjects

The present study comprised 167 healthy adults (age: 33.4 ± 12.6 years, 77 males) with normally directed LV rotational mechanics. Subjects were recruited for screening between 2011 and 2015 on a voluntary basis. A physical examination, a laboratory test, standard 12-lead electrocardiography (ECG), and two-dimensional Doppler echocardiography (2DE) were performed, and the results were in the normal reference ranges in all subjects. No one used any drugs or was a smoker or obese. Subjects with a known disease, pathological state, or clinical conditions that could affect the results were excluded from the study. Three-dimensional speckle-tracking echocardiography (3DSTE)-derived data acquisition was performed together with 2DE in accordance with recent guidelines and practices, and detailed 3DSTE-derived analysis was performed later offline [1,2,3,4]. The present retrospective cohort study was part of the study named ‘Motion Analysis of the heart and Great vessels bY three-dimensionAl speckle-tRacking echocardiography in Healthy subjects’ (MAGYAR-Healthy) Study, which was organized at the University of Szeged [1]. It was partly aimed at the physiological analysis of 3DSTE-derived variables including LV and LA volumetric and functional parameters (‘Magyar’ means ‘Hungarian’ in the Hungarian language). The study was conducted in accordance with the Declaration of Helsinki (as revised in 2013). The Institutional and Regional Human Biomedical Research Committee of University of Szeged, Hungary (No.: 71/2011) approved the study. All subjects participating in the study provided informed consent.

### 2.2. Two-Dimensional Doppler Echocardiography

In the case of all healthy individuals, the complete routine 2DE evaluation of cardiac chambers and valves was performed, including chamber quantifications, the exclusion of significant valvular regurgitations or stenoses with Doppler echocardiography, and the Doppler evaluation of early- (E) and late- (A) diastolic mitral inflow velocities and their ratio (E/A) in accordance with recent guidelines. A Toshiba Artida^TM^ echocardiographic system (Toshiba Medical Systems, Tokyo, Japan) attached to a PST-30BT (1–5 MHz) phased-array transducer was used for all examinations [9].

### 2.3. Three-Dimensional Speckle-Tracking Echocardiography

For the 3DSTE studies, the same Toshiba Artida^TM^ echocardiographic machine (Toshiba Medical Systems, Tokyo, Japan) was used, attached to a PST-25SX matrix transducer. Firstly, subjects in sinus rhythm were lying in left lateral position; then, 3D echocardiographic data were acquired during a breath hold from the apical window. For optimal images, 6 subvolumes were acquired within 6 cardiac cycles, which were merged together automatically for a full-volume 3D echocardiographic dataset. Detailed offline data analysis was performed with vendor-provided 3D Wall Motion Tracking software version 2.7 (Ultra Extend, Toshiba Medical Systems, Tokyo, Japan). Data acquisitions were performed by the same observer (ÁK) with many years of experience in 3DSTE [1,2,3,4].

### 2.4. 3DSTE-Derived Determination of LV Twist

**Figure 1:**
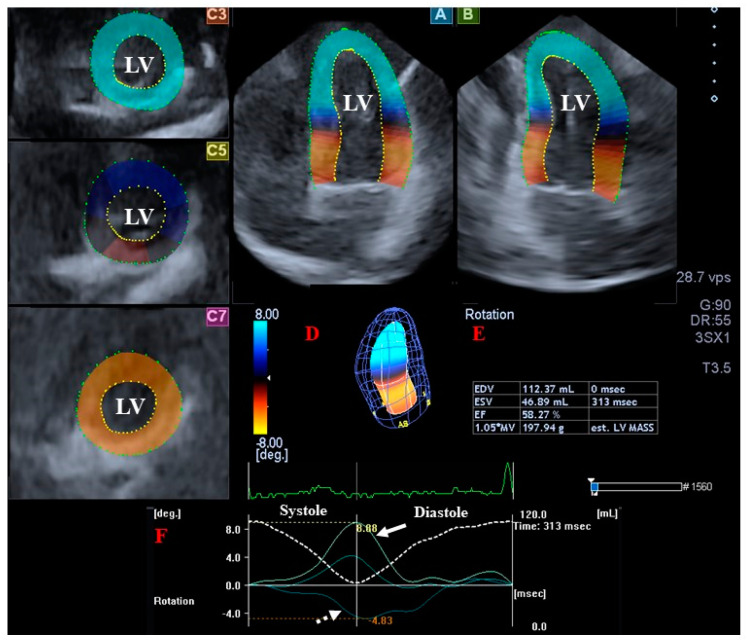
Assessment of the left ventricle (LV) by three-dimensional (3D) speckle-tracking echocardiography. Apical four-chamber view (**A**); apical two-chamber view (**B**); and short-axis views at basal (**C3**), midventricular (**C5**), and apical LV levels (**C7**) are presented together with a 3D LV cast (**D**) and calculated LV volumetric data (**E**). The LV apical rotation curve (white arrow) and LV basal rotation curve (dashed white arrow) are also presented, together with the LV volume change curve during the cardiac cycle (dashed white line) (**F**). Abbreviations: LA, left atrium; LV, left ventricle; RA, right atrium; RV, right ventricle; EDV, end-diastolic volume; ESV, end-systolic volume; EF, ejection fraction.

For the quantification of LV rotational mechanics, optimal apical longitudinal views including apical longitudinal 4-chamber (AP4CH) and 2-chamber (AP2CH) views and basal, midventricular, and apical cross-sectional planes were selected, and mitral annular (MA)-LV edges and the endocardial surface of the LV apex were defined. Then, a virtual 3D cast of the LV was created after sequential analysis (Figure 1). According to our practices, the following features of LV rotational mechanics were determined from the same LV 3D model [10,11]:

- Clockwise basal LV rotation (in degrees);
- Counterclockwise apical LV rotation (in degrees);
- LV twist (net difference between LV apical and basal rotations in degrees);
- Time-to-peak LV twist (in milliseconds).

In the event that the direction of the LV apical and basal rotations was the same (clockwise/counterclockwise), LV twist could not be realized (absent), and only an LV apico-basal gradient was present. This situation is called LV ‘rigid body rotation’ (RBR) [12]. Subjects with LV-RBR were not involved in this study.

### 2.5. 3DSTE-Derived Determination of LA Volumes

**Figure 2:**
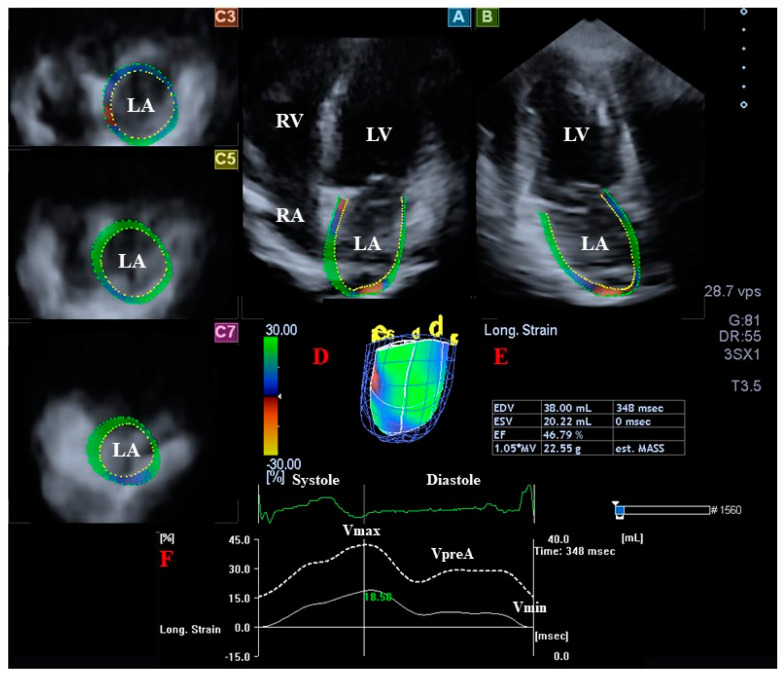
Assessment of the left atrium (LA) by three-dimensional (3D) speckle-tracking echocardiography. Apical four-chamber view (**A**); apical two-chamber view (**B**); short-axis view at basal (**C3**), midatrial (**C5**), and superior left atrial level (**C7**) are presented together with a 3D model of the LA (**D**) and calculated LA volumetric data (**E**). LA time—global volume change (dashed white line) and global longitudinal strain change (white line) during the cardiac cycle are also presented (**F**), together with maximum (Vmax), pre-atrial contraction (VpreA), and minimum (Vmin) LA volumes. Abbreviations: LA, left atrium; LV, left ventricle; RA, right atrium; RV, right ventricle; EDV, end-diastolic volume; ESV, end-systolic volume; EF, ejection fraction; Vmax, end-systolic maximum LA volume; VpreA, early-diastolic pre-atrial contraction LA volume; Vmin, end-diastolic minimum LA volume.

Similarly to LV analysis, longitudinal AP4CH and AP2CH views and 3 short-axis views in the basal, midatrial, and superior regions were selected for LA assessments (Figure 2). A 3D cast of the LA was created after setting multiple reference points in AP4CH and AP2CH, starting at the lateral edge of the MA-LA and proceeding toward the LA apex to the edge of the septal MA-LA. The LA appendage and the pulmonary veins were excluded from the assessments. Following LA border detection at end-diastole, similarly to LV, a sequential analysis was performed. The following LA volumes were determined using this LA 3D cast according to the cardiac cycle [13,14]:

- Vmax—end-systolic maximum LA volume, measured just before mitral valve opening (largest LA volume);
- VpreA—LA volume before atrial contraction in early diastole, at the time of P wave on ECG;
- Vmin—late diastolic minimum LA volume, measured just before mitral valve closure (smallest LA volume).

Then, the following LA functional properties were calculated from the LA volumes:

End-systolic reservoir LA function

- TASV—LA total stroke volume, calculated by Vmax − Vmin;
- TAEF—LA total emptying fraction, calculated by total SV/Vmax.

Early-diastolic conduit LA function

- PASV—LA passive stroke volume, calculated by Vmax − VpreA;
- PAEF—LA passive emptying fraction, calculated by passive SV/Vmax.

Late-diastolic booster pump (active contraction) LA function

- AASV—LA active stroke volume, calculated by VpreA − Vmin;
- AAEF—LA active emptying fraction, calculated by active SV/VpreA.

### 2.6. Statistical Analysis

Continuous and categorical variables are presented as mean ± standard deviation (SD) or in number/percentage formats. A *p*-value less than 0.05 was considered to be significant. Independent sample *t*-test and analysis of variance (ANOVA) were used for comparison among the groups, where appropriate. Statistical analyses were performed with SPSS software version 22 (SPSS Inc., Chicago, IL, USA).

## 3. Results

### 3.1. Clinical and Two-Dimensional Doppler Echocardiographic Data

According to routine 2DE data, the LA diameter measured via the parasternal long-axis view (36.7 ± 4.0 mm); LV end-diastolic diameter (48.1 ± 3.7 mm) and volume (107.0 ± 22.9 mL); LV end-systolic diameter (40.4 ± 25.5 mm) and volume (36.6 ± 9.3 mL); and interventricular septum (9.0 ± 1.5 mm), LV posterior wall (9.1 ± 1.6 mm), and LV ejection fraction (65.9 ± 4.9%) were in the normal range. The mean E/A proved to be 1.38 ± 0.35. None of the healthy adults showed larger than grade 1 valvular regurgitation or valvular stenosis on any valves.

### 3.2. Classification of Subjects

The mean ± SD of the 3DSTE-derived parameters of healthy subjects are presented in Table 1. Healthy subjects were classified into three groups according to the normal Vmax, VpreA, and Vmin and apical and basal LVrots: the estimated mean ± SD served as the lower (27.8 mL, 15.9 mL, 11.3 mL, 5.4°, and 2°, respectively) and upper (54 mL, 39.5 mL, 27.5 mL, 13°, and 6.2°, respectively) values.

### 3.3. 3DSTE-Derived LA Volumes and LV Rotational Parameters

**Figure 3:**
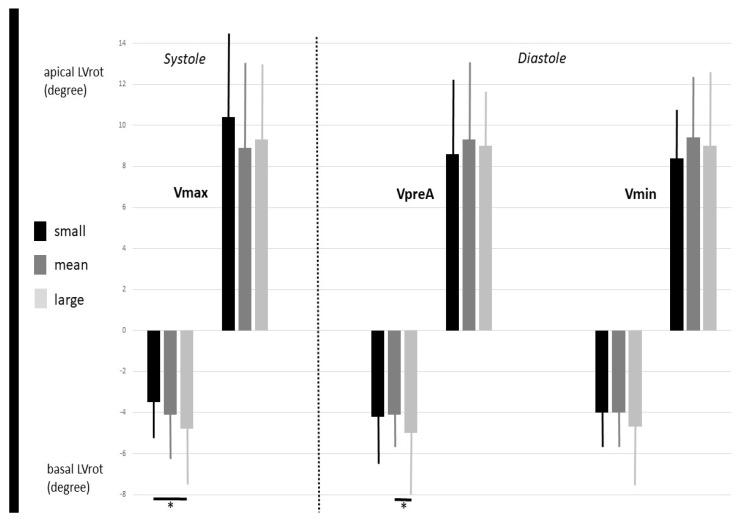
The dependence of the apical and basal left ventricular rotations on increasing left atrial volumes. Abbreviations: LVrot, left ventricular rotation; Vmax, end-systolic maximum left atrial volume; VpreA, early-diastolic pre-atrial-contraction left atrial volume; Vmin, end-diastolic minimum left atrial volume. Bold black lines represent significant difference between subgroups. * *p* < 0.05 between groups.

The mean frame rate of the measurements was 28.3 ± 1.1 vps., while artifact incidence in the 3D image acquisition was observed in 22 out of 167 cases (13%). None of the subjects had LV-RBR. An increased end-systolic Vmax was associated with increased early- (VpreA) and end- (Vmin) diastolic LA volumes, and all stroke volumes were increased as well. TAEF did not change with increasing Vmax, while PAEF was reduced and AAEF was increased when the Vmax was highest. An increasing early-diastolic VpreA was associated with increasing Vmax and Vmin and TASV and AASV but was not associated with PASV. Moreover, an increasing VpreA showed associations with a reduction in TAEF and PAEF. The highest AAEF was seen at the highest VpreA. Vmin showed similar associations with atrial volumes, stroke volumes, and emptying fractions. The systolic basal LVrot was the highest in the case of the highest systolic Vmax and early-diastolic VpreA. The apical LVrot did not show obvious associations with any increasing LA volumes (Table 2, Figure 3).

**Figure 4:**
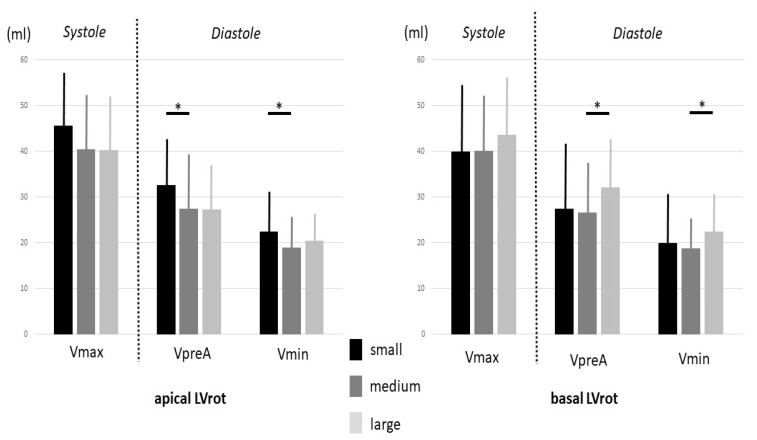
The dependence of left atrial volumes on increasing apical and basal left ventricular rotation. Abbreviations: LVrot, left ventricular rotation; Vmax, end-systolic maximum left atrial volume; VpreA, early-diastolic pre-atrial-contraction left atrial volume; Vmin, end-diastolic minimum left atrial volume. Bold black lines represent significant difference between subgroups. * *p* < 0.05 between groups.

The highest systolic basal LVrot was associated with significantly increased VpreA and Vmin. TAEF and PAEF were the lowest when the basal LVrot was the highest. Reduced diastolic LA volumes (VpreA, Vmin) were seen in cases of increased apical LVrot. TAEF was the lowest when the apical LVrot was the highest. AASV and AAEF showed reductions in cases of increased apical LVrot. An increasing basal LVrot was associated with the tendentious lowering of the apical LVrot and the significant elevation of LV twist. Similarly, increasing apical LVrot was associated with the tendentious lowering of the basal LVrot and the significant elevation of LV twist (Table 3, Figure 4).

### 3.4. Correlations

No correlation could be demonstrated between any left atrial volumes and volume-based functional properties and the LV rotational parameters.

## 4. Discussion

The main finding of the present study was that the end-systolic basal LVrot was the highest when the end-systolic Vmax and early-diastolic VpreA were the highest. Similar findings for end-systolic apical LVrot could not be detected. Interestingly, the highest end-systolic basal LVrot was associated with a significantly increased diastolic VpreA and Vmin, while lower diastolic LA volumes were seen in cases of increased end-systolic apical LVrot. A possible interpretation of this result is that when a larger blood volume flows into the LV during diastole, an increased LVrot is first detected in systole in the basal LV region close to the LV inflow/LA outflow mitral annulus. End-systolic apical LVrot was associated with lower LA volumes, suggesting that it plays a role in lower atrial preload.

Moreover, an increased basal LVrot was associated with tendentiously lowered apical LVrot and significantly elevated LV twist. Similarly, increased apical LVrot was associated with tendentiously lowered basal LVrot and significantly elevated LV twist. These findings could suggest an interplay between the basal and apical LV regions in optimizing the efficiency of LV rotational mechanics.

There is increasing interest in the non-invasive clinical assessment of the physiologic relationships between aorta, cardiac chambers, and valves, due to the fact that recently developed cardiovascular imaging techniques allow their detailed analysis, including the evaluation of their effects on each other. In the last few decades, significant developments in echocardiography have resulted in the 3DSTE, which encompasses the advantages of 3D echocardiography and STE, including accurate volumetric measurements and the quantification of segmental/regional/global wall contractility features throughout the cardiac cycle, achieved from a single acquisition of all cardiac chambers [1,2,3,4]. However, the LV is also able to rotate around its long axis due to the muscle bands running perpendicular to each other in the endocardium and epicardium, creating a form of movement similar to the wringing of a towel, which significantly increases the effectiveness of this contractility that is responsible for 40% of ejection [5,6]. This sort of movement is called an LV twist and is based on the counterclockwise rotation of the LV apical segments and the clockwise rotation of the LV basal segments [5,6]. Three-dimensional speckle-tracking echocardiography is the first non-invasive imaging technique with the ability to quantify LV rotational mechanics [10,11]. The 3DSTE-derived determination of LV rotational mechanics has been validated [15,16,17], and the normal reference values for LV rotational parameters are well-defined [10]. Although LV rotational mechanics play a significant part in LV function, their prognostic significance has not been confirmed [1,2,3,4].

The relationship between myocardial preload and mechanical performance is described by the Frank–Starling law, which has been demonstrated for the LV [18] and LA [19,20] but has not been clearly identified in the right atrium [21]. The Frank–Starling law describes the ability of the heart (and the LV) to change the wall contraction force and therefore the stroke volume in response to changes in venous return (and atrial preload). A higher preload is associated with a higher force and contractility. However, the relationship between the atrial preload and LV rotational mechanics has never been assessed by 3DSTE, a method which is capable of not only detecting LV rotational parameters, but also conducting detailed LA volumetric assessments throughout the cardiac cycle, making use of a 3D echocardiographic dataset acquired at the same time [22,23,24]. Three-dimensional speckle-tracking echocardiography-derived LA volumes have been validated [22,23,24], and normal LA volumetric reference values have been determined [13].

The associations presented above showed the significant adaptation of the LV rotational mechanics to increased LA preload even in healthy circumstances, suggesting the significant role of the LV rotational mechanics in the physiological response to elevated LA volumes. The clinical implications of these results suggest that clinical practitioners should be aware of the importance of LV rotational mechanics and their dependence on LA volumes during the cardiac cycle. In principle, these adaptations are more prevalent in several disorders involving changes in LA volume. Therefore, further studies are warranted to confirm these results using other methods and involving a larger number of healthy adults and patients with different disorders experiencing volumetric changes.

## 5. Limitations

The most important limitations of this study are listed below:

- If 2DE and 3DSTE are compared in terms of image quality, 2DE has significant benefits, which limits the usability of 3DSTE [1,2,3,4]. Therefore, further developments are essential to improve the spatial and temporal resolution of 3DSTE.
- Although 3DSTE is suitable for simultaneous LV strain assessments using the same 3D echocardiographic dataset, a detailed analysis of these parameters (LV strains and LA volumes) would have surpassed the limits of this publication and is a topic for another paper demonstrating the dependence of LV deformation on LA volumetric changes [1,2,3,4].
- Similarly, LA volumes and strains could be measured from the same acquired datasets [14,20], although they also have effects on each other [20].
- The present study did not aim to validate 3DSTE-derived LV rotational and LA volumetric parameters, as this has already been achieved [15,16,17,22,23,24].
- This study had a retrospective study design, and so associations were only established between risk factors and outcomes, and not between causes and effects.

## 6. Conclusions

Strong associations and adaptations between 3DSTE-derived LA volumes throughout the cardiac cycle and LV rotational mechanics were observed, even in healthy circumstances.

## Figures and Tables

**Table 1:** Three-dimensional speckle-tracking echocardiography-derived left atrial volumetric and left ventricular rotational parameters.

Parameters	Data
**Left atrial volumes**	
maximum left atrial volume (V_max_, mL)	40.9 ± 13.1
pre-atrial-contraction left atrial volume (V_preA_, mL)	27.7 ± 11.8
minimum left atrial volume (V_min_, mL)	19.4 ± 8.1
total atrial stroke volume (TASV, mL)	21.5 ± 8.2
total atrial emptying fraction (TAEF, %)	52.6 ± 11.9
passive atrial stroke volume (PASV, mL)	13.1 ± 5.7
passive atrial emptying fraction (PAEF, %)	33.1 ± 12.7
active atrial stroke volume (AASV, mL)	8.3 ± 5.8
active atrial emptying fraction (AAEF, %)	29.0 ± 11.9
**Left ventricular rotational mechanics**	
basal left ventricular rotation (basal LV_rot_, °)	−4.1 ± 2.1
apical left ventricular rotation (apical LV_rot_, °)	9.2 ± 3.8
left ventricular twist (LV_twist_, °)	13.3 ± 4.1
time-to-LV_twist_ (ms)	346 ± 132

**Table 2:** Left atrial volumes and left ventricular rotational parameters in different left atrial volume groups.

	V_max_ < 27.8 mL (*n* = 25)	27.8 mL ≤ V_max_ ≤ 54.0 mL (*n* = 116)	54 mL < V_max_ (*n* = 26)	V_preA_ < 15.9 mL (*n* = 18)	15.9 mL ≤ V_preA_ ≤ 39.5 mL (*n* = 125)	39.5 mL < V_preA_ (*n* = 24)	V_min_ < 11.3 mL (*n* = 23)	11.2 mL ≤ V_min_ ≤ 27.5 mL (*n* = 123)	27.5 mL < V_min_ (*n* = 21)
V_max_ (mL)	23.9 ± 3.6	38.8 ± 6.4 ^‡^	64.8 ± 7.0 ^‡,‡‡^	25.6 ± 5.8	38.7 ± 8.5 *	63.2 ± 9.6 *,**	26.4 ± 5.4	39.7 ± 9.2 ^†^	62.7 ± 10.8 ^†,††^
V_preA_ (mL)	16.0 ± 4.2	25.6 ± 7.0 ^‡^	47.5 ± 11.2 ^‡,‡‡^	12.9 ± 2.5	25.4 ± 6.0 *	50.5 ± 8.3 *,**	14.5 ± 3.5	26.5 ± 7.7 ^†^	49.3 ± 9.0 ^†,††^
V_min_ (mL)	11.9 ± 3.7	18.2 ± 5.5 ^‡^	31.3 ± 8.7 ^‡,‡‡^	9.1 ± 1.8	18.2 ± 4.9 *	33.1 ± 7.8 *,**	9.1 ± 1.7	18.5 ± 4.2 ^†^	35.6 ± 5.5 ^†,††^
TASV (mL)	12.0 ± 3.1	20.6 ± 5.5 ^‡^	33.4 ± 7.6 ^‡,‡‡^	16.5 ± 5.0	20.6 ± 7.0 *	30.0 ± 9.9 *,**	17.3 ± 4.6	21.2 ± 8.0 ^†^	27.1 ± 9.1 ^†,††^
TAEF (%)	50.5 ± 12.6	53.1 ± 11.8	51.8 ± 11.6	63.3 ± 7.9	52.4 ± 11.6 *	46.9 ± 12.1 *,**	64.7 ± 7.2	52.2 ± 11.1 ^†^	42.3 ± 9.4 ^†,††^
PASV (mL)	7.9 ± 2.9	13.2 ± 4.9 ^‡^	17.2 ± 7.1 ^‡,‡‡^	12.7 ± 4.4	13.3 ± 5.9	12.7 ± 5.8	12.0 ± 4.4	13.2 ± 5.8	13.3 ± 6.6
PAEF (%)	33.5 ± 12.8	34.5 ± 12.4	27.2 ± 12.4 ^‡‡^	48.4 ± 8.7	33.7 ± 11.4 *	19.7 ± 7.9 *,**	44.5 ± 11.4	33.0 ± 11.6 ^†^	20.8 ± 9.1 ^†,††^
AASV (mL)	4.1 ± 2.9	7.3 ± 3.6 ^‡^	16.2 ± 8.6 ^‡,‡‡^	3.8 ± 2.0	7.3 ± 3.4 *	17.4 ± 8.4 *,**	5.4 ± 3.2	8.0 ± 5.6 ^†^	13.8 ± 6.1 ^†,††^
AAEF (%)	24.9 ± 14.6	28.5 ± 10.9	33.3 ± 13.3 ^‡,‡‡^	28.3 ± 12.9	28.3 ± 11.3	33.8 ± 13.9 **	34.7 ± 14.3	28.5 ± 11.7 ^†^	27.1 ± 8.4 ^†^
basal LV_rot_ (°)	−3.5 ± 1.8	−4.1 ± 2.0	−4.8 ± 2.7 ^‡^	−4.2 ± 2.0	−4.0 ± 1.9	−5.0 ± 3.0 **	−4.0 ± 1.9	−4.0 ± 1.9	−4.7 ± 3.0
apical LV_rot_ (°)	10.4 ± 4.1	8.9 ± 3.7	9.3 ± 3.7	8.6 ± 3.5	9.3 ± 3.9	9.0 ± 3.7	8.4 ± 3.0	9.4 ± 3.9	9.0 ± 3.6
LV_twist_ (°)	13.9 ± 3.6	13.1 ± 4.2	14.1 ± 3.9	12.8 ± 3.5	13.2 ± 4.2	14.0 ± 4.3	12.4 ± 3.1	13.3 ± 4.1	13.7 ± 4.2
time-to-LVtwist (ms)	371 ± 146	340 ± 133	349 ± 108	372 ± 146	339 ± 132	355 ± 116	334 ± 131	342 ± 134	366 ± 115

* *p* < 0.05 vs. VpreA < 15.9 mL; ^†^ *p* < 0.05 vs. Vmin < 11.3 mL; ^‡^ *p* < 0.05 vs. Vmax < 27.8 mL; ** *p* < 0.05 vs. 15.9 mL ≤ VpreA ≤ 39.5 mL; ^††^ *p* < 0.05 vs. 11.2 mL ≤ Vmin ≤ 27.5 mL; ^‡‡^ *p* < 0.05 vs. 27.8 mL ≤ Vmax ≤ 54.0 mL.

**Table 3:** Left atrial volumes and left ventricular rotational parameters in different left ventricular apical and basal rotation groups.

	Basal LV_rot_ < 2° (*n* = 21)	2° ≤ Basal LV_rot_ ≤ 6.2° (*n* = 121)	6.2° < Basal LV_rot_ (*n* = 25)	Apical LV_rot_ < 5.4° (*n* = 24)	5.4° ≤ Apical LV_rot_ ≤ 13.0° (*n* = 116)	13.0° < Apical LV_rot_ (*n* = 27)
V_max_ (mL)	39.8 ± 14.4	40.1 ± 12.3	43.5 ± 13.6	45.5 ± 11.4	40.4 ± 13.4	40.2 ± 12.9
V_preA_ (mL)	27.2 ± 14.8	26.6 ± 10.2	32.0 ± 13.3 ^†^	32.5 ± 13.0	27.3 ± 12.1 ^#^	27.2 ± 10.4
V_min_ (mL)	19.9 ± 10.7	18.5 ± 7.3	22.4 ± 8.2 ^†^	22.4 ± 8.6	18.9 ± 8.1 ^#^	20.4 ± 8.4
TASV (mL)	19.9 ± 6.4	21.6 ± 8.4	21.2 ± 7.9	23.1 ± 6.2	21.5 ± 8.3	19.8 ± 8.7
TAEF (%)	51.3 ± 11.4	53.8 ± 12.1	48.5 ± 11.2 ^†^	51.6 ± 11.0	53.5 ± 11.4	48.6 ± 13.9 ^‡^
PASV (mL)	12.5 ± 5.0	13.6 ± 6.0	11.5 ± 4.8	13.0 ± 5.1	13.1 ± 5.7	13.0 ± 6.4
PAEF (%)	33.8 ± 14.2	34.3 ± 12.3	28.0 ± 12.4 ^†^	30.2 ± 13.7	33.4 ± 12.5	32.4 ± 13.9
AASV (mL)	7.4 ± 5.1	8.1 ± 5.5	9.6 ± 6.8	10.1 ± 6.4	8.5 ± 6.0	6.8 ± 4.4 ^#^
AAEF (%)	26.0 ± 10.7	29.6 ± 12.3	28.3 ± 10.6	30.3 ± 10.8	29.9 ± 11.8	24.0 ± 11.7 ^#‡^
basal LV_rot_ (°)	−1.29 ± 0.65	−3.74 ± 1.12 *	−7.97 ± 1.27 *^†^	−4.29 ± 2.65	−4.22± 2.05	−3.60 ± 1.80
apical LV_rot_ (°)	9.46 ± 4.21	9.29 ± 3.79	8.26 ± 3.67	3.55 ± 1.52	8.73 ± 2.05 ^#^	15.37 ± 1.93 ^#‡^
LV_twist_ (°)	10.76 ± 4.37	13.04 ± 3.81 *	16.23 ± 3.97 *^†^	7.84 ± 3.12	12.95 ± 2.70 ^#^	18.98 ± 2.54 ^#‡^
time-to-LVtwist (ms)	289 ± 107	356 ± 142 *	342 ± 86	326 ± 148	349 ± 131	345 ± 108

* *p* < 0.05 vs. basal LVrot < 2°; ^†^ *p* <0.05 vs. 2° ≤ basal LVrot ≤ 6.2°; ^‡^ *p* < 0.05 vs. 5.4° ≤ apical LVrot ≤ 13.0°. ^#^ *p* < 0.05 vs. apical LVrot < 5.4°.

## Data Availability

The data are not publicly available due to ethical reasons.
